# Canine *Staphylococcus argenteus*: Case Report from The Netherlands

**DOI:** 10.3390/pathogens11020153

**Published:** 2022-01-26

**Authors:** Eelco F. J. Meijer, Anne van Renssen, Ianthe Maat, Linda van der Graaf-van Bloois, Birgitta Duim, Els M. Broens

**Affiliations:** 1 Department of Medical Microbiology and Radboud Center for Infectious Diseases, Radboud University Medical Center, 6525 GA Nijmegen, The Netherlands; ianthe.maat@radboudumc.nl; 2 Orthopedics Department, Medical Centre for Animals, 1105 AZ Amsterdam, The Netherlands; orthopeed@mcvoordieren.nl; 3 Department of Biomolecular Health Sciences, Faculty of Veterinary Medicine, Utrecht University, 3584 CL Utrecht, The Netherlands; L.vanderGraaf@uu.nl (L.v.d.G.-v.B.); B.Duim@uu.nl (B.D.); E.M.Broens@uu.nl (E.M.B.)

**Keywords:** *Staphylococcus argenteus*, canine, genome sequencing, ST2250

## Abstract

*Staphylococcus argenteus* has been reported worldwide in humans, while reported non-human cases are sparse. Its complete epidemiology, alongside its infectivity and pathogenicity in humans and non-humans, remain to be clarified. Here, we describe the first reported canine *Staphylococcus argenteus*, causing a deep wound infection in a Labrador retriever after orthopedic surgery. The closed genome is reported, with phylogenic and genetic analyses, as well as extensive phenotypic antimicrobial susceptibility testing for human and veterinary antibiotics. No genetic explanation could be found for its interaction with a canine host, underscoring the intrinsic multispecies pathogenicity and potential (anthropo-)zoonotic spread of *Staphylococcus argenteus*.

## 1. Introduction

*Staphylococcus argenteus* has recently been identified as a novel coagulase-positive species within the *Staphylococcus aureus* complex lacking the carotenoid pigment staphyloxanthin, formerly described as the distinct *S. aureus* lineage clonal complex 75 [1]. Several more clonal complexes have been described to belong to *S. argenteus* since and multiple studies have elaborated on its worldwide spread, including Australia, Africa, Asia, America and Europe [2]. *S. aureus* and *S. argenteus* have been implicated in disease in humans and non-humans [2,3], whereas the closely related *S. schweitzeri* has been found primarily in bats and non-human primates and no human infections have been described to date [4,5]. Human *S. argenteus* infections are associated with serious morbidity, mortality and nosocomial infection in humans [3,6], and isolates have been shown to harbor most of the virulence genes of *S. aureus* [5]. Non-human described cases include gorilla [7], cow [8], rabbit [9], and pig [10]. However, gaps in knowledge on the epidemiology, infectivity and pathogenicity of *S. argenteus* in humans and non-humans remains to be clarified. In the Netherlands, human cases have been described [3]. Here, we describe the first reported canine *S. argenteus*.

## 2. Case

A 9-year-old female Labrador retriever underwent a Tibia Plateau Levelling Osteotomy (TPLO) procedure after cranial cruciate ligament rupture in October 2019. She had a wound infection after 6 days under oral amoxicillin/clavulanic acid (12.5 mg/kg) prophylaxis, appearing superficial at first with increasing wound exudate and subsequent purulent drainage, without systemic symptoms. The wound infection was then empirically treated with oral doxycycline (10 mg/kg) for 10 days with success, allowing the wound to close. However, the wound reopened spontaneously 13 days after antibiotic treatment cessation and bacterial cultures were taken, after which targeted oral enrofloxacin (5 mg/kg) was prescribed for 14 days after positive cultures for *Corynebacterium auris*. Three weeks later, the wound infection persisted and a deep infection was suspected. Arthrocentesis was performed, with peroperative deep bacterial cultures yielding both *Pseudomonas aeruginosa* and *Staphylococcus argenteus*. The veterinary clinic decided not to treat the enrofloxacin-sensitive *Pseudomonas aeruginosa*. Oral doxycycline (10 mg/kg) was reinitiated to treat the *S. argenteus* infection, for another 3 weeks, with treatment success.

## 3. Materials and Methods

**Figure 1:**
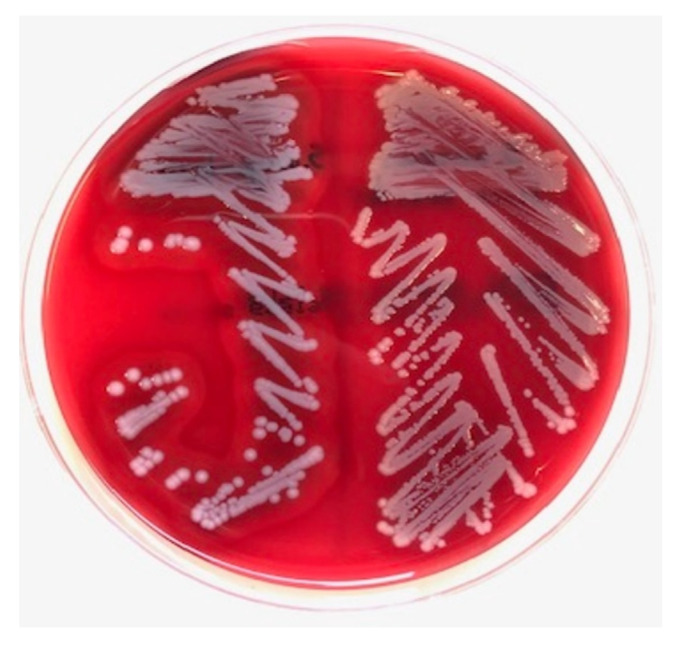
Colony morphology of found *Staphylococcus argenteus* (left), showing creamy white colonies with a β-hemolytic zone on sheep blood agar. *Staphylococcus aureus* (right) for comparison.

Bacterial cultures were performed on sheep blood agar plates (bioTRADING Benelux B.V., Mijdrecht, the Netherlands). *Staphylococcus* spp. was suspected based on colony morphology in routine diagnostics. The *S. argenteus* colony morphology appeared as creamy white ‘argent’ colonies with a β-hemolytic zone (Figure 1). Overnight colony material was smeared on an MSP 96 target, and 1 uL of 70% formic acid was added, followed by addition of 1 uL of MALDI matrix solution (Bruker Corporation, Billerica, MA, USA). The MALDI-TOF MS (Bruker Corporation, Billerica, MA, USA) analyses were performed in duplicate and resulted in the identification of *S. argenteus* with scores of 2.19 and 2.26.

**Figure 2:**
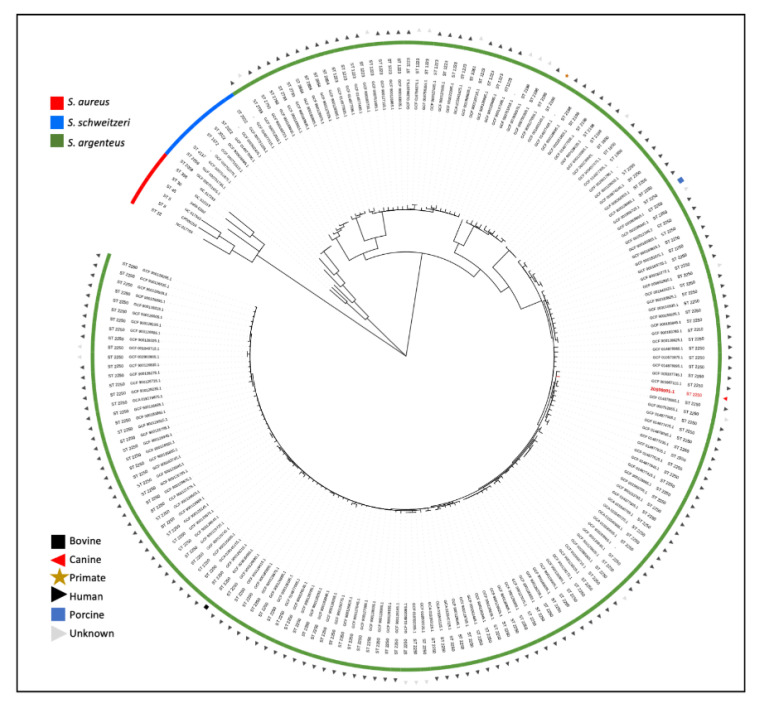
Phylogenetic tree of the core genome SNPs diversity of *Staphylococcus argenteus*, *Staphylococcus schweitzeri* and *Staphylococcus aureus*. The identified *S. argenteus* ST2250 isolate 20S00001-1 is indicated in red. Branch lengths in the tree were adjusted to sites per core genome and square root transformed to enhance resolution. All publicly available *S. argenteus* and *S. schweitzeri* isolates were included. Available *S. aureus* reference isolates of the most common *S. aureus* clonal complexes were used for rooting.

DNA was isolated using the DNeasy Ultra Clean Microbial kit (Qiagen, Venlo, The Netherlands) and was not sheared and size-selected for sequencing. For whole-genome sequencing, an Illumina library was prepared using the Nextera kit and pooled libraries were sequenced using Nextseq 500 (Illumina, San Diego, CA, USA). Nanopore long-read sequencing was performed according to protocol (SQK-LSK109) on a MinION device (Oxford Nanopore Technologies, Oxford Science Park, UK). Hybrid assembly was performed using Unicycler v0.4.9b [11], and the reads and complete assembled genome of isolate 20S00001-1 are available from the European Nucleotide Archive browser at http://www.ebi.ac.uk/ena/browser/view/GCA_927312875 (accessed on 25 January 2022). Using in silico multilocus sequence typing (MLST) [12], the strain was identified to belong to sequence type ST2250. Using AMRFinderPlus [13], two likely chromosomal resistance genes were identified; *tet(38)* and *fosB*. For phylogenetic analysis (Figure 2), a tree based on single-nucleotide polymorphisms (SNPs) in the core genome was constructed using the genome alignment made with parsnp v1.2 [14] with filtering of recombination regions using gubbins v 2.3.4. [15]. All available *S. argenteus* and *S. schweitzeri* isolates from the MLST database (accessed on 13 July 2021, https://pubmlst.org/organisms/staphylococcus-aureus/) were included. Publicly available *S. aureus* reference isolates of the most common *S. aureus clonal complexes*, sequence types ST5, ST8, ST22, ST30 and ST45, were used for rooting [16].

Using the virulence factor database [17] for this isolate, genes were found related to adherence virulence factors including autolysins (*atl*), adhesins (*ebh, ebp, eap, efb, fnbA/B, sdrC-H* and *spa*) and adhesins critical for biofilm formation (*icaA-C,R*). Staphylocoagulase (*coa*) and thermonuclease (*nuc1*) were identified. Genes related to immune evasion were *scn* and *sbi*, as well as several genes responsible for exoenzyme and exotoxin secretion with the Type VII secretion system (*esaA,B; esaG; essA-C* and *esxA-D*) and multiple undetermined capsule genes. Toxins included hemolysins (*hla, hld, hlgA-C*), multiple undetermined exotoxins, staphylococcal enterotoxins and enterotoxin-like toxins (*sec, selq*), and leukocidins (*lukD*). Panton–Valentine leucocidin (*lukF/S*) and toxic shock syndrome toxin (*tst/tsst*) were negative.

For phenotypic detection of resistance, a combination of agar diffusion with automated antibiogram (BD Phoenix system; Becton, Dickinson and Company, Franklin Lakes, NJ, USA) and microdilution (custom-made microdilution plates; MERLIN Diagnostika GmbH, Bornheim, Germany) were used for human and veterinary antibiotics, respectively. Fosfomycin MIC was verified by Etest (BioMérieux, Marcy l’Etoile, France). Antibiogram methods used are common practice and used in clinical diagnostics. EUCAST (human) [18] and CLSI (veterinary) [19] clinical breakpoints were applied; results are listed in Table 1. Phenotypic antibiotic resistance was identified for fosfomycin.

## 4. Discussion

Here, we describe the first canine *S. argenteus* reported to date. Colony morphology, coagulase testing and 16S rRNA sequencing cannot distinguish *S. argenteus* from *S. aureus*, but an updated MALDI-TOF MS database can reliably identify *S. argenteus* profiles that are present in the database [20]. MALDI-TOF MS and whole-genome sequencing are currently regarded as valid diagnostic tools [2], while only the latter can provide definite identification [1]. For routine identification of the canine isolate, MALDI-TOF MS and subsequent whole-genome sequencing were employed. The reported canine *S. argenteus* isolate genetically belongs to the most frequent isolated lineage, ST2250 [5].

Because of its recent discovery [1] and limited known genetical subtypes, it is currently unclear whether *S. argenteus* has been endemic worldwide or recently spread globally [5]. This will be a challenge to address with the current problematic delimitation of *S. argenteus* from *S. aureus* in routine diagnostics and the relatively high costs of whole-genome sequencing. There are also no known clinical consequences of misidentifying *S. argenteus* as *S. aureus* due to their seemingly comparable pathogenicity. In the Netherlands, multiple methicillin-resistant *S. argenteus* have been identified retrospectively from human clinical and outpatient settings [3]. Two patients had likely imported the isolates from hospitals in Australia and Philippines—known geographical ‘hot spots’ of *S. argenteus*- [2]—while others had an unknown source. Regarding methicillin resistance in *S. argenteus*, routine phenotypic and genotypic approaches for *S. aureus* are applicable, and infection prevention and control measures are equal.

*S. argenteus* has been reported to harbor a multitude of virulence factors common in well-characterized *S. aureus* lineages [2,5,21], associated with *S. aureus* pathogenicity and causing a similar spectrum of disease. Comparing virulence genes identified in the canine isolate with neighboring human isolates within the phylogenetic tree, no unique genes were identified, thus no genetic explanation could be found for its interaction with a canine host. In fact, this isolate had fewer virulence genes and toxins when compared with the isolates found in humans, comparable to the isolate found in a gorilla [7]. Interestingly, from all genomes shown in Figure 2, two closely related genomes (GCF_003967115.1 and GCF_014877235.1) harbored the *tsst-1* gene coding for toxic shock syndrome toxin-1. Panton–Valentine leucocidin (*lukF/S*) and toxic shock syndrome toxin (*tst/tsst*) were, however, not identified in this isolate.

The *fosB* and *tet(38)* genes identified in this *S. argenteus* isolate did phenotypically confer resistance to fosfomycin but not tetracycline (Table 1). Doxycycline resulted in treatment success, as was expected with proven tetracycline susceptibility. *Tet38* is a known chromosomally encoded native efflux pump in *S. aureus*, which can confer resistance if (additionally) plasmid encoded [22]. The *fosB gene* also does not always confer phenotypic resistance [23,24] and is postulated to influence cellular processes, including adaptation to reactive oxygen species [25], while the drug fosfomycin is also known to be naturally produced by *Streptomyces* spp. [26] and *Pseudomonas* spp. [27]. An environmental origin can therefore not be excluded. However, fosfomycin is a drug commonly used in human medicine and *fosB* resistance genes have been commonly reported in *S. aureus* clinical methicillin-resistant *S. aureus* (MRSA) strains, although rare in methicillin-susceptible *S. aureus* (MSSA) [28]. Recently, *fosB* resistance genes were found in canine methicillin-resistant *Staphylococcus pseudintermedius* (MRSP) isolates [24] and in several coagulase-negative staphylococci in colony-born and wild vervet monkeys on the small island of Saint Kitts [29]. Fosfomycin is rarely used in veterinary medicine, which raises questions regarding the source of the *fosB* gene in animal isolates. The presence of *fosB* hypothetically might benefit survival due to its effects on cellular processes, rather than antibiotic resistance. Interestingly, in a recent genomic analysis of 132 known global *S. argenteus* strains, about 90% of the isolates were found to carry highly similar, likely chromosomally encoded *fosB* genes [23]. Taken together, *fosB* is commonly found in *S. argenteus*, but we cannot confer environmental, human or non-human origin.

The *S. argenteus* ST2198 and ST2250 found in animals are abundantly found in humans (Figure 2). The relative lack of virulence factors and antibiotic resistance in the found canine isolate, whilst causing disease early postoperatively, underscores its intrinsic pathogenicity. As doxycycline resulted in treatment success, this argues in favor of the clinical relevance of *S. argenteus* in this patient. It was unclear how the dog acquired the isolate, but the lack of travel history suggests this strain was acquired locally. Foodborne transmission has been described for *S. argenteus* [30], but the dog did not receive raw foods. Unfortunately, due to loss of follow-up, no swabs were taken from household members to evaluate *S. argenteus* colonization. Specific history from household members could not be taken, but they did not report any skin and soft tissue infections at the time of veterinary visit.

## 5. Conclusions

We report the first canine *S. argenteus* isolate, undoubtedly locally acquired, belonging to the most frequent isolated lineage ST2250. Using whole-genome sequencing, we found clear overlap in virulence genes with *S. aureus*. Resistance to fosfomycin was identified both phenotypically and genetically, but no genetic explanation could be found for its interaction with a canine host. Our finding underscores its multispecies pathogenicity and its potential (anthropo-)zoonotic spread.

## Figures and Tables

**Table 1:** Phenotypic detection of resistance. A combination of agar diffusion with automated antibiogram (BD Phoenix system; Becton, Dickinson and Company, Franklin Lakes, NJ, USA) and microdilution (custom-made microdilution plates; MERLIN Diagnostika GmbH, Bornheim, Germany) were used. Fosfomycin MIC was verified by Etest (BioMérieux, Marcy l’Etoile, France). ^§^ EUCAST clinical breakpoints were applied, http://www.eucast.org (accessed on 25 January 2022) [18]. ^‡^ CLSI breakpoints were applied for bacteria isolated from animals, https://clsi.org (accessed on 25 January 2022) [19].

Antibiotic	MIC or Disk Zone	Interpretation
** Agar diffusion (mm) **		
Cefoxitin	29	Susceptible ^§^
Clindamycin	28	Susceptible ^§^
Rifampicin	32	Susceptible ^§^
Trimethoprim	26	Susceptible ^§^
** Automated antibiogram (mg/L) **		
Ceftaroline	0.5	Susceptible ^§^
Chloramphenicol	8	Susceptible ^§^
Ciprofloxacin	≤0.5	Susceptible ^§^
Daptomycin	0.5	Susceptible ^§^
Erythromycin	≤0.2	Susceptible ^§^
Fusidic acid	≤0.5	Susceptible ^§^
Gentamycin	≤1	Susceptible ^§^
Levofloxacin	≤0.5	Susceptible ^§^
Linezolid	2	Susceptible ^§^
Moxifloxacin	≤0.25	Susceptible ^§^
Mupirocin	≤0.5	Susceptible ^§^
Penicillin	0.125	Susceptible ^§^
Quinupristine/Dalfopristine	≤0.5	Susceptible ^§^
Teicoplanin	≤0.5	Susceptible ^§^
Tetracycline	≤0.5	Susceptible ^§^
Tigecycline	≤0.125	Susceptible ^§^
Tobramycin	≤1	Susceptible ^§^
Trimethoprim/sulfamethoxazole	≤0.5	Susceptible ^§^
Vancomycin	1	Susceptible ^§^
** Microdilution (mg/L) **		
Enrofloxacin	≤0.25	Susceptible ^‡^
Kanamycin	≤16	Susceptible ^‡^
Neomycin	≤8	Susceptible ^‡^
** Epsilometer test (mg/L) **		
Fosfomycin	64	Resistant ^§^

## Data Availability

The reads and complete assembled genome of *S. argenteus* isolate 20S00001-1 are available from the European Nucleotide Archive browser at http://www.ebi.ac.uk/ena/browser/view/GCA_927312875 accessed on 25 Januanry 2022.
